# Predicting school uptake of The Daily Mile in Northern Ireland- a data linkage study with School Census Data and Multiple Deprivation Measures

**DOI:** 10.1371/journal.pone.0294648

**Published:** 2023-12-14

**Authors:** Gavin Breslin, Medbh Hillyard, Noel Brick, Stephen Shannon, Brenda McKay-Redmond, Mark Shevlin, Barbara McConnell

**Affiliations:** 1 School of Psychology, Queens University Belfast, Belfast, Northern Ireland; 2 Bamford Centre for Mental Health and Wellbeing, School of Psychology, Ulster University, Coleraine, Northern Ireland; 3 School of Psychology, Ulster University, Coleraine, Northern Ireland; 4 Sport and Exercise Sciences Research Institute, Ulster University, Belfast, Northern Ireland; 5 Early Childhood Studies Department, Stranmillis University College, Belfast, Northern Ireland; Aichi Prefectural Mikawa Aoitori Medical and Rehabilitation Center for Developmental Disabilities, JAPAN

## Abstract

**Background:**

Participating in physical activity benefits health, yet a majority of children remain inactive. The Daily Mile^™^ (TDM) originated in Scotland in 2012 with the aim of increasing primary school children’s physical fitness. Despite being a practically feasible and popular initiative, it remains unclear the extent to which schools implement TDM, and whether TDM core principles are adhered to (i.e., run or jog *at least* 3-days per week). In Northern Ireland it is unknown how many schools regularly participate in TDM, and whether there is an association between TDM participation with school type, school location, size, total number of children attending the school, school deprivation level, and/or motivation as measured by the COM-B model (Capabilities, Opportunities, Motivation model of behaviour). Therefore, this study aimed to quantify the uptake of TDM in Northern Ireland, assess whether schools are following the core principles, and analyse if there is an association between aforesaid demographic factors and TDM participation.

**Methods:**

An online cross-sectional survey was sent to all primary and special education schools in Northern Ireland with the support of the Education Authority for Northern Ireland and the Public Health Agency for Northern Ireland. The survey was completed by the school principal or teacher, and was available from 31^st^ August until 16^th^ December 2022. Survey results were linked with the 2021/2022 Northern Ireland School Census Data and Northern Ireland Multiple Deprivation Measure 2017. Quantitative and qualitative questions were included in the survey to assess participation and implementation of TDM.

**Results:**

The survey received 609 school responses. After data cleaning, and removal of duplicates from schools a sample of 358 primary schools (45%) and 19 special education schools (47.5%) was analysed. Over half (54.7%) of primary schools and 36.8% of special education schools reported taking part in TDM. More special education needs schools reported taking part in their own version of an ‘active mile’ rather than TDM formally, and qualitative findings showed TDM was not perceived as appropriate for many children in special educational settings. There was wide variation in adherence to TDM core principles. A multivariate binary logistic regression model was fitted to the data, but it was not statistically significant (χ^2^(17) = 22.689, p = .160). However, univariate effects showed that increasing levels on COM-B (Capability) was associated with increased likelihood of TDM participation (OR = 2.506), and Catholic Maintained schools were almost twice as likely as Controlled schools to be delivering TDM (OR = 1.919). There was no association found between deprivation and TDM uptake.

**Conclusion:**

Encouragingly over 50% of schools in Northern Ireland reported taking part in TDM. However, despite being a low-cost and practically feasible physical activity initiative, further intervention work with sound research methodology is needed to promote adherence to TDM core principles to maximise benefits to children’s health. Furthermore, concerted efforts are required to adjust TDM so that it is inclusive for all educational settings, and children’s abilities.

## Background

The benefits of physical activity (PA) for children’s physical and psychological well-being have been well documented [1], including helping to maintain a healthy weight status [2], improving physical fitness [3], enhancing mental health [4] and cognitive function [1]. Despite the espoused health benefits, uptake of, and adherence to, the United Kingdom (UK) Chief Medical Officers’ physical activity guidelines for children of at least 60 minutes of Moderate-to-Vigorous intensity PA (MVPA) each day has remained low, and often worsened over the past four decades [5]. For example, in the UK, approximately 20–45% of 5- to 16-year-olds meet these recommendations [6], while in Northern Ireland 20% of primary school aged children (4–11-year olds) met the PA guidance [7]. As most children spend large amounts of time at school, the school setting is deemed a suitable and opportune location to increase PA levels for the improvement of health. In addition, school pupils who have historically displayed lower PA levels, or have limited access and opportunities for PA, such as children from socio-disadvantaged backgrounds, can be targeted for intervention [8, 9].

While after-school sport activities offer some promise to curbing physical inactivity, such programmes usually incur a financial cost, and are time and resource intensive. Consequently, creative and practically feasible means to boost activity during the school day are needed [10]. Some further potential barriers to implementing school-based PA interventions pertain to the typical requirements for teachers, or external facilitators, to be skilled and knowledgeable in implementing PA, and the requirement for complex equipment and/or extensive PA facilities. A programme that is less equipment and human resource intensive is The Daily Mile (TDM). TDM started in a primary school in Scotland in 2012 and has since been widely adopted by schools worldwide in an attempt to increase the PA and fitness levels of primary school children [11]. TDM premise is that children should run or jog outdoors for 15 minutes (approximately one mile) on at least three days of the week. TDM aims to be simple and quick to implement, without increasing teacher’s workload, or significantly impeding curriculum time within the school day. TDM has 10 core principles: (1) It should be quick, taking just 15 minutes with no requirement to set up equipment (2) It should be fun for the children (3) 100% inclusive for all children’s abilities and school circumstances (4) The weather should not be a barrier (5) The route should be firm and mud free (6) A risk assessment of the route should be carried out (7) Children should take part in TDM during curriculum time and should do TDM at least 3 times a week (8) Children should do TDM in their uniform, with no need to change into PE kit (9) Able-bodied children should jog or run for the full 15 minutes (10) Schools should try and keep TDM simple [11].

To date over 17,000 schools from 90 countries worldwide are signed up to TDM events [11]. Registration with TDM foundation has been described as an intention to adopt TDM rather than a proxy measure of participation in TDM [12]. In Northern Ireland (NI), approximately 412 primary schools (51%) are registered with the Daily Mile Foundation [11], but some schools reported merely registering for a one-off event. Therefore, it remains unclear to what extent NI schools are completing TDM regularly in line with the core principles. NI is a jurisdiction with complex social, political, and economic circumstances wherein schools and communities are often segregated on religious grounds, and a legacy of violent conflict referred to as ‘the troubles’ has had stark implications for healthcare and education [13–15]. NI children’s PA compares unfavourably with their counterparts in Great Britain and the Republic of Ireland, and hospital waiting lists are among the highest in the UK. As such, NI represents a region in particular need of health prevention initiatives through physical activity including TDM [16].

Supporting the positive effects of TDM participation, evidence has shown that children who perform TDM three or more times a week have reported improvements in physical fitness and some aspects of mental health, including self-perception, cognitive function, and healthier weight status [17]. Larger effects have been observed with completing TDM three or more times per-week in line with TDM core principles, compared to 2-times per week, highlighting a case for careful consideration of TDM fidelity [18]. To this end, a recent systematic review of the health benefits of TDM outlined that compliance with the core principle of 3-days per-week or more is likely to reduce over time, and despite early reported positive health benefits, such outcomes may diminish without sufficient adherence [17].

Understanding physical activity from a socio-ecological perspective accounts for individual-level variables (e.g., PA beliefs, motivations to be active) to broader social, physical, and policy considerations [19], and is essential for overcoming barriers and facilitating enabling factors in interventions [20]. Regarding TDM, there is scant research generally, and even less so in NI, on whether TDM uptake and adherence is reliant on socio-ecological factors such as school type, location, or deprivation levels. A recent data linkage study in England found variations in school characteristics in TDM uptake [12]. For example, the authors reported that those schools participating in TDM had a larger mean number of pupils, were likely to be in urban rather than rural areas and had higher levels of socially disadvantaged pupils [12]. Furthermore, despite TDM principle of inclusivity [11], there is limited research with children with intellectual or physical disabilities. Such participants/schools have been excluded from trials or analysis [18, 21–23]. The exception was one study in which children were recruited from special education schools and non-special educational needs schools [24]. However, as the data from both school groups were presented together, it was not possible to differentiate the effects on special educational needs children alone [24]. To overcome the lack of much needed social and demographic information on TDM’s implementation, a similar analysis as Venkatraman et al.’s [12] in NI is warranted.

One further consideration of intervention efficacy is the lack of a theoretical foundation to why TDM can increase PA and fitness. In consideration of psychosocial factors related to TDM school uptake and adherence, TDM was pragmatically developed without a formal theoretical underpinning, and this atheoretical foundation may limit the identification and control of variables that can help guide long-term sustainability of TDM within the school environment. As such, the integration of a programme theory to support TDM and better understand children and staff’s engagement has been suggested [25]. As a starting point, the capability, opportunity, motivation model of behaviour (COM-B), has been validated and tested to explain PA behaviour by the interaction of individual, social and environmental factors [26]. Within the COM-B model, Capability focuses on the perceived psychological and physical capability of an individual to engage in a physical activity, including having the required skills and knowledge. Opportunities relate to all the external factors which can enable the behaviour to happen, such as physical space and geography of the school grounds. Motivation covers both reflective and automatic motivation and is explained in terms of the psychological processes which help to initiate a behaviour [26]. Developing COM-B survey questions at a school level can help establish if differences exist between TDM schools and non-TDM schools and offer theoretical insight to barriers and facilitators of PA in children.

Hence, the aim of the present study was to conduct a cross-sectional survey of primary and special educational needs schools to determine the prevalence of TDM in NI. A second aim was to assess adherence to TDM core principles. The survey was designed to measure how many schools take part in TDM, the level of fidelity in TDM schools, and barriers to implementation and maintenance of TDM. Finally, we explored the predictors of TDM uptake (or not) in schools through several variables consistent with a socio-ecological multilevel statistical model [19]. Variables tested in the model were: the school having a Physical Education (PE) Co-ordinator, senior staff having PE or sport qualifications, COM-B factors of Capability, Opportunity and Motivation. As well as the total number of children enrolled in the school, total percentage of free school meals, total percentage of newcomer pupils (A pupil who does not have satisfactory language skills to participate fully in the school curriculum and does not have a language in common with the teacher [27]), school management type (Catholic Maintained; Controlled; Other), Urban/Rural, Income deprivation, health and disability deprivation, education, skills and training deprivation, income deprivation affecting children and County of school.

## Methods

### Design

A cross-sectional survey was sent to all primary and special education schools in Northern Ireland. The survey was completed online using the survey platform Qualtrics (Qualtrics, Provo, UT). Email addresses for all schools were extracted from the Department of Education Institution Search (http://apps.education-ni.gov.uk/appinstitutes/default.aspx). Schools were emailed with details of the study and a link to the survey.

### Setting and sample size

Northern Ireland has a population of 1.9 million people [28]. There are 784 primary schools in Northern Ireland for 4–11-year-olds, under various management types; controlled, Catholic maintained, other maintained, controlled integrated and grant maintained integrated. There are an additional 12 preparatory schools which also cater for 4–11-year-olds. For statistical analysis and reporting primary and preparatory schools data were combined. There are also 40 Special Educational Schools in Northern Ireland which cater for children ranging from 3–19 years old with various educational needs and abilities.

### Respondents

The primary or special educational needs school Principal or teacher with most knowledge on the school’s participation in TDM were requested to complete the survey.

### Procedures

The Survey was available for online completion from 31^st^ August 2022 until 16^th^ December 2022. An email with details of the study and a link to the survey was sent out to all primary and special education needs schools in Northern Ireland. To increase participation details of the research and a link to the survey were also shared in relevant private Facebook groups, through various health and school networks and on The Daily Mile Network Northern Ireland Twitter account. Follow up emails were also sent, and the survey was shared by both the Education Authority for Northern Ireland and the Public Health Agency for Northern Ireland. Schools that had not completed the survey were then sent a letter in the post and schools who were still yet to complete the survey were then followed up with a phone call. If a teacher or Principal was uncontactable, a voice message was left for the Principal asking them to complete the survey. The full survey is available on request.

### Measures

#### Demographic

School demographic details collected were: School name and postcode; PE coordinator; staff members with PE or Sport qualifications.

#### The Daily Mile

Participation in the TDM was assessed by asking respondents to select one of 4 options in response to the following question: “Is your school currently delivering… (1) The Daily Mile (2) Your own version of an ‘active mile’ (3) None (4) None currently but previously took part in The Daily Mile”. TDM schools were subsequently asked if they were signed up to The Daily Mile on the Daily Mile Foundation website and to report the date of signing up (if applicable).

#### Core principles

TDM schools adherence to core principles were assessed via a series of questions, for example “When does The Daily Mile most commonly take place?” The responses were either: 1. During curriculum time, 2. At break/lunchtime or 3. Before/after school.

#### Non-TDM schools

The barriers to continued participation in TDM schools who had previously participated but had stopped were determined through questions on reasons for stopping TDM. In addition, non-TDM schools were asked to select barriers to signing up to TDM. Free text responses to questions on barriers to signing up were not analysed thematically but were used to highlight examples.

#### COM-B

General attitudes to physical activity in both TDM schools and non TDM schools were assessed by asking for levels of agreement with 10 statements based on the capability, opportunity and motivation model of behaviour (COM-B) [26]. Questions were developed based upon work by McDermott et al. [29] and the Sport England Active Lives Surveys [9]. Four questions focused on capabilities, three on opportunities and three on motivation (see Table 3 in *Results*). For example, questions asked whether: ‘Children in our school would be more physically active if they had more knowledge on how to be active’ (COM-B Capability), ‘Children in our school would be more physically active if we had access to better facilities’ (COM-B Opportunity), ‘Children in our school would be more physically active if they were set goals and targets in relation to the amount of activity they do at school’ (COM-B Motivation). Cronbach’s alpha was calculated for each of the three domains and indicated good internal reliability for the capabilities subscale: (Cronbach’s alpha, 0.80, 95% CI 0.76–0.83), and questionable reliability for the opportunities subscale (Cronbach’s alpha, 0.62, 95% CI 0.55–0.69), and questionable reliability for the motivation subscale (Cronbach’s alpha, 0.68, 95% CI 0.62–0.74). The Cronbach’s alpha score for the ten statements combined was 0.81 (95% CI 0.78–0.84) indicating good internal reliability for the combined scale.

### Data sources

A database was created linking the survey data with data obtained from the Northern Ireland School Census 2021/22 and data from The Northern Ireland Multiple Deprivation Measure (NIMDM) 2017 [30]. In both cases, the 2021/2022 School Census and the 2017 NIMDM represented the most recently available data to include within this database.

The Northern Ireland School Census takes place annually in October and is a count of all children attending schools in Northern Ireland. The census collects data on a broad range of areas [31]. The fields used in this study were: percentage of children receiving Free School Meals (FSM), percentage of newcomer children, number of pupil enrolments and school management type (i.e., controlled, Catholic maintained, other maintained, controlled integrated and grant maintained integrated). In Northern Ireland there are various different types of primary schools for 4–11 year olds. Controlled schools are managed by the Education Authority (EA) through Boards of Governors, the Board consists of representatives mainly from the Protestant Churches, along with representatives of parents. Catholic Maintained Schools are managed by Boards of Governors which consist of members nominated by Trustees (mainly Roman Catholic), along with representatives of parents, teachers, and the EA. Schools within the ‘Other’ category include Controlled and Grant Maintained Integrated Schools. Integrated Schools include children from different cultures and religious beliefs (including reasonable numbers of both Protestant and Roman Catholic children), those from different socio-economic backgrounds and those of different abilities [31]. The selected fields (excluding school management type) were chosen based upon the study by Venkatraman et al. [12].

NIMDM 2017 ranks the 890 Super Output areas (SOAs) in Northern Ireland from the most deprived (rank 1) to the least deprived (rank 890) [30]. The multiple deprivation score is calculated by combining the weighted scores for seven domains, the seven domains are: Income deprivation, Employment Deprivation, Health Deprivation and Disability, Education, Skills and Training Deprivation, Access to Services, Living Environment and Crime and Disorder [30]. As well as the Multiple deprivation measure, this study used the income deprivation measure, health deprivation and disability, education skills and training deprivation and income deprivation affecting children.

### Data analyses

The name of the schools was recorded during data collection, however this was removed before analysis to anonymise the schools. Frequencies and percentages were calculated for categorical variables (i.e., Delivering TDM, PE co-ordinator, PE qualifications, location (urban/rural), school management type, agreement with COM-B statements, TDM foundation registrations, year groups participation, adherence to core principles). Means and standard deviations were calculated for continuous variables (i.e., Total enrolment, % free school means, % Newcomer pupil, NIMDM). Chi-squared tests were used to test the association between school demographics and delivering TDM or not. Multivariate binary logistic regression was carried out to determine if there was an association between school characteristics, deprivation measures, agreement with COM-B statements, the county the school was located and participation in TDM. For the purpose of the regression analysis the statements related to the 3 elements of the COM-B model were grouped into three categories (e.g. capabilities, opportunities and motivation). All quantitative analyses were conducted using Statistical Package for the Social Sciences Version 26. Statistical significance was set to p<0.05. The data was cleaned and analysed by MH with the support of GB and MS. A statistician (MS) carried out the multivariate binary logistic regression.

### Ethical considerations

Ethical approval was obtained from the University’s School of Psychology Research Ethics Filter Committee. When participants clicked the survey link a study information sheet was provided outlining the aims of the study. Participants provided opt-in consent. It was not possible for participants to begin the survey without providing consent.

## Results

There are 784 primary schools and 12 preparatory schools, totalling 796 schools in Northern Ireland for 4–11-year-olds. There are an additional 40 special education needs schools which support education for a range of age groups. A total of 609 responses were received for the survey (i.e., 77% of total). Once duplicates and entries from wrong school types (nurseries and pre-schools) were removed, a total sample of 358 (45%) primary/preparatory schools and 19 (47.5%) special education needs schools remained. Results for primary and special education needs schools are reported separately.

### Primary schools

#### Primary schools taking part in TDM

Out of the 358 included primary schools, 54.7% reported taking part in TDM and 31% reported taking part in other ‘Active Miles’ rather than TDM. There was no statistically significant difference between TDM schools and non-TDM schools with regards to urban/rural locations (54% rural for both). There was a slightly lower percentage of children on free school meals in TDM schools compared to non-TDM schools (26% vs 28%, respectively). TDM schools had a higher percentage of newcomer children than non-TDM schools (13% vs 11%) and TDM schools had a larger mean number of pupils compared to non-TDM schools (255 vs 249), however neither difference was statistically significant. There was a lower percentage of controlled schools in TDM group (39.8%) compared to the non-TDM group (52.5%). There was also a higher percentage (p = .03) of Catholic maintained schools in the TDM group (52.6%), compared to non-TDM schools (38.3%) (see Table 1).

**Table 1:** Demographics of primary schools.

	All primary schools mean (SD) (n = 358)	TDM schools (n = 196)	Non TDM schools (n = 162) (including ‘Active Mile’ schools)	Northern Ireland
**Size**				
Total enrolments (No. of pupils)	252.31 (176.08)	255.44 (175.67)	248.52 (177.05)	216.49
Physical Education (PE) Co-ordinator	85.5%	86.2%	85.2%	-
Staff with PE qualifications	42.9%	43.9%	41.6%	-
**Location**				
Rural	53.6%	53.6%	53.7%	55.7%
Urban	46.4%	46.4%	46.3%	44.3%
% Free School meals	26.83 (15.82)	26.15 (15.09)	27.66 (16.67)	28.3
% Newcomer children	12.08 (13.68)	13.03 (15.64)	10.92 (10.85)	7.3
**Type of School**				
Controlled	45.5%	39.8%*	52.5%*	44.6%
Catholic Maintained	46.1%	52.6%*	38.3%*	44.6%
Other	8.4%	7.7%	9.3%	10.8%

Note:

*Indicates a statistically significant difference (p < .05)

### Current daily mile primary schools

#### Registration with TDM foundation

Sixty-six percent of TDM schools reported being registered as a participating school on TDM foundation website, 26% of schools were unsure if they were signed up and 8% of schools said they were not registered. Most schools have registered from 2017 onwards, with 40% of schools having signed up since 2020.

#### Implementation according to TDM core principles

TDM schools were scored according to how many of the core principles they followed (see Table 2). Although TDM Foundation highlights ten core principles underpinning TDM, adherence to the core principles ‘route’ and ‘simple’ were not included due to assessment difficulties, and the core principle ‘when’ was split into two for analysis purposes as it includes both when pupils should complete TDM and frequency per week it should be completed. Therefore, the highest obtainable score was nine and the lowest score was zero (if none of the core principles were followed). Only one school (0.5% of sample) scored nine points. The lowest score obtained was three. The mean score was 6.5 (SD 1.21). The core principles most commonly followed were ‘clothes’ (children run in their clothes without changing into kit), ‘when to go’ (children do TDM during curricular time) and ‘100%’ (TDM is fully inclusive and children with mobility difficulties are supported to take part). The core principles which were least followed were own pace (1%), weather not a barrier (69.8%) and frequency (70.8%). There was no significant association between number of core principles followed and length of time since daily mile registration.

**Table 2:** Adherence to core principles.

Core Principle	% Following core principle
Clothes- children should run in school clothes without changing into kit	91.1%
When to go- during curricular time	91.1%
100%- Always fully inclusive	84.1%
Fun- It should be fun for the children	82.8%
Quick- It should take just 15 minutes	82.3%
Risk- Risk assessment of route carried out	75%
Frequency- Minimum of 3 times a week	70.8%
Weather–Weather not a barrier	69.8%
Own pace- done properly it is not a walk, able-bodied children should aim to run or jog for full 15 minutes	1%

#### Year groups

Sixty-seven percent (67%) of schools reported that all year groups took part in TDM. One school reported that only one year took part (Primary 6 ages 9–10). In 14.4% of schools some year groups took part and 18% reported most year groups taking part. In the schools which reported that most/some years took part the most common classes taking part were Primary 4–7 (ages 7–11-year-olds).

#### Sustaining enthusiasm and participation

Schools selected which factors that they perceived were the most important for sustaining enthusiasm and participation in TDM. The two most selected responses were consistent scheduling (TDM performed at the same time each day) and embedding TDM within the school culture (for example, staff engaging in TDM and running with the children).

#### COM-B model

All respondents were asked for their level of agreement on whether the children in their school would be more active based on a series of statements centred around the COM-B model of behaviour change (see Table 3). Overall, the statements which had the highest level of agreement were that children would be more active if they had greater support from parents/family (83.5%), there was more time in the school day (83%), and they had access to better facilities (80.3%). Levels of agreement with the statements on capabilities were higher in TDM schools and levels of agreement with opportunity statements were higher in non TDM schools, however these differences were not statistically significant.

**Table 3:** Agreement with COM-B model statements.

COM-B element	Statement		% Strongly Agree/Agree		
		All	TDM schools	Non TDM schools	P Value
Capability	They had a better understanding of why PA is important	70.9	74.5	67.1	.124
Capability	They had more knowledge on how to be active	73.7	76	70.8	.266
Capability	They were physically stronger	54.5	59.2	48.7	.050
Capability	They had more physical stamina	76	78.9	72.5	.163
Opportunity	There was more time to include PA into the school day	83	81.6	84.6	.462
Opportunity	We had access to better facilities	80.3	79	82	.476
Opportunity	The weather was better	66.7	66.2	67.3	.822
Motivation	They received greater support to be active from staff	50.4	49	52.2	.548
Motivation	They received greater support to be active from parents/family	83.5	84.2	82.7	.709
Motivation	They were set goals and targets in relation to the amount of activity they do at school	75.4	74	77.2	.487

#### Multivariate binary logistic model

To address the third study aim, a multivariate binary logistic regression model was fitted to the data. The dependent variable was whether a school was currently delivering TDM or not. The independent variables were: PE co-ordinator- Y/N, PE Qualifications-Y/N, COM-B Capability (scale score), COM-B Opportunity (scale score), COM-B Motivation (scale score), Total Enrolment, Free school meals (%), Newcomer pupil (%), School management type (Catholic Maintained; Controlled; Other), Urban/Rural, Income deprivation, Health and disability, Education, skills and training, Income dep affecting children and County.

All variables were entered into the model, but it was not statistically significant (χ^2^(17) = 22.689, p = .160). However, the univariate effects showed that greater levels of agreement on the COM-B capability statements were associated with increased likelihood of daily mile participation (OR = 2.506). In addition, Catholic maintained schools were almost twice as likely as controlled school to deliver TDM (OR = 1.919); this likely became non-significant in the multivariate model as school type was associated with other predictor variables such as urban-rural and percentage of free school meals.

#### Non-daily mile primary schools

Schools who were not signed up to TDM reported various barriers to signing up. The three most cited were not enough time in the school day (35%), adverse weather (27%) and no access to suitable space in the school grounds (14%). Some of the qualitative free text responses highlighted other problems associated with implementing TDM, such as perceiving it “Difficult to accommodate so many children in such a small space”, “Difficult to timetable into school day with small staff numbers” and “difficult to get buy in from staff members”.

#### Previous daily mile primary schools

A total of 35 schools (21.6% of sample) reported having previously taken part in TDM but subsequently stopping. The most frequently given reasons for stopping were that it took up too much time (25%), poor weather (25%) and teacher’s workload (23%).

#### Special education needs schools

Nineteen of 40 (47.5%) special education needs schools completed the survey. Seven schools (36.8%) reported taking part in TDM, eight schools, (42.1%) were doing their own version of an active mile, three schools (15.8%) reported not taking part in TDM or other active mile, and one school (5.3%) reported previously taking part in TDM but was not currently.

#### Implementing TDM according to core principles in special education schools

Five schools (71%) reported TDM was carried out as a walk, two schools reported pupils ran and walked. Two out of the seven schools reported all children took part. With regards to frequency of TDM, five schools reported carrying out TDM every day, with two schools performing it three or four times a week.

The most commonly reported reason for schools not signing up was that it was not appropriate for the needs of the majority of their pupils. Staff gave reasons such as “We are a SLD (Specific Learning Disability) school for pupils 3–8 years old, the majority of our pupils could not do this”, “Not appropriate for all SEN pupils”, “Very few of our pupils would be capable of walking a mile” “Due to the physical disabilities of our children it is not possible/appropriate for us to take part”. Other commonly cited barriers were not having access to a suitable space to do TDM and increasing teachers’ workload.

## Discussion

This study explored the uptake and participation of TDM in schools in Northern Ireland. We examined demographic and theoretically derived COM-B predictors of school participation in TDM whilst assessing fidelity (how it was implemented) and barriers of TDM implementation in schools. As TDM has been adopted rapidly into schools, community settings and some government policy, it was important to develop an understanding of the socio-ecological factors predicting the uptake of TDM, and therefore better inform future school, community and health promotion policy-approaches [17].

From a representative sample, 54.7% of primary schools reported taking part in TDM in NI. This adoption rate of 54.7% is slightly lower than in a study in Leicester where there was a 59.5% uptake [32], but higher than reported in London where the adoption rate was reported as 48% [33]. In our study, 31% of primary schools reported taking part in other ‘Active Miles’ rather than TDM. Our study found there was a significant variability in how schools conducted TDM, and whether TDM’s ten core principles were followed. Given the preliminary evidence of TDM’s benefits for children’s health [17], it is recommended that TDM is performed on a minimum of three days of the week. Whilst encouragingly 71% of primary schools in Northern Ireland reported taking part in TDM on at least three days of the week, this figure is considerably lower than in Leicester, where 96% of schools reported taking part in TDM on at least three days of the week [32]. Previous studies have reported that in some schools during busy periods (e.g., assessments, assembly, open days, visitors to the school) the frequency of TDM may be reduced or when the curriculum was considered ‘too full’ [33]. Primary schools in Northern Ireland have a shorter school day with P1-P3 (ages 4–7) being in school for approximately five hours per day compared to children of the same age in England being in school for approximately six and a half hours per day. A shorter day and earlier finish for children may make it more practically difficult to fit TDM into the school day and may be one factor to explain the lower percentage of primary schools completing TDM in Northern Ireland on at least three days of the week compared to figures reported in England. Another factor which may explain the lower frequency of participation in Northern Ireland, is the transfer test for grammar school selection, which takes place in Term one of Primary 7 (10–11 year olds). Schools typically spend a lot of time in Primary 6 and 7 preparing pupils for the transfer test and anecdotal evidence would suggest Primary 6/7 teachers commonly stop or reduce the frequency of TDM during this time, along with other non-curricular activities. Frequency of TDM participation is important to gain maximum health benefits as children who complete it more often (3x/week or more) have demonstrated greater increases in fitness than those participating twice a week [18].

In NI, TDM principle with the lowest reported compliance was pace/intensity of the activity. The Daily Mile Foundation specify that the TDM should be performed at a jog or run by able-bodied children and not performed as a walk. The intensity at which TDM is performed may be important from the perspective of the children’s PA guidelines for health, specified as at least 60 minutes daily of moderate to vigorous intensity activity, which is presumably achieved through a jog or run for most children [34]. Only two schools reported TDM being performed as a jog or run, with most schools saying it was completed by both running and walking (81%) and 18% of schools reported doing TDM as a walk. The intensity at which TDM is performed will likely impact the potential physical health outcomes for the children. However, the issue balancing the trade-off between maximising health benefits with the affective experience to exercise remains highly debateable. In short, while increased health benefits are associated with higher intensity physical activities, there is greater potential for these activities to feel unpleasant for most individuals, which may negatively influence the subsequent likelihood of engaging in that physical activity behaviour [35]. Rather, individuals have various preferences for physical activity intensities, and lower intensity PA (e.g., walking) has many complementary physical and mental health benefits and may engage a broader spectrum of individuals to adhere to physical activity, with the proposal that physical activity messaging should focus on the view that *‘doing some activity is better than none’* [36]. Therefore, given that children and young people are unlikely to be proficient in regulating their PA intensities with their affective responses, it would appear that a strict prescription of jogging or running TDM may be discouraged from the perspectives of varying children’s fitness levels. Instead, a more inclusive focus on offering children autonomy over what intensity and mode of aerobic behaviour they prefer to practice through TDM may strike a better balance between maximising both health benefits and likelihood of adherence [35]. In a broader sense, there is limited evidence available to draw comparisons within schools performing TDM in regular conditions and not as part of a trial on TDM. In a systematic review of TDM, six studies reported TDM should be performed at a run or walk and only one study reported that children were asked to run or jog [17]. Other studies have reported a large degree of variability of the intensity TDM is performed at with the most active children spending the full duration of TDM at moderate intensity PA, which would indicate they were running or jogging, compared to the least active children who only spent 33% of TDM at moderate intensity PA [23].

Another factor which has appeared to cause some confusion with the name ‘Daily Mile’ is whether or not children should complete a full mile whilst participating. Studies have shown that some teachers focus on children completing a mile whereas others defined their initiative as 15 minutes of activity [37]. In our current study over 80% of schools reported TDM lasting 15 minutes, however 12% of schools were spending longer than the recommend 15 minutes. Again, there is a need for clear and consistent messaging that TDM is intended to be fifteen minutes of jog or running activity and maybe not all children will complete a mile within that time.

Qualitative research exploring teachers’ experiences of TDM found that schools implement TDM differently, with some schools adopting a rigid approach, whereas others felt flexibility around the core principles was important to fit into their school setting [38] and although TDM is intended to be simple to implement and not need any additional equipment, schools have often reported the need to integrate rewards, games and competition, in order to maintain interest and motivation [33, 37]. Our survey found that the most common responses to what factors were important to sustain enthusiasm and participation in TDM was embedment into the school culture (i.e., an ethos of active health and wellbeing). Qualitative research supports this finding with teachers having reported that they found their participation in TDM motivated children to keep going [33, 37].

The results of the current study showed that schools in Northern Ireland that participated in TDM had higher levels of agreement with the COM-B capability statements, suggesting that knowledge and understanding around physical activity is important to increase participation in physical activities such as TDM. As a result, consideration needs to be given on how best to increase capabilities within the non-TDM schools. One approach would be to offer advice and guidance on the benefits of physical activity and the simplicity of TDM implementation. In England, Scotland and Wales there are Daily Mile co-ordinators, who work to support schools in implementing TDM and focusing on delivering it according to the core principles, however, Northern Ireland is the only part of the UK that does not currently have a Daily Mile co-ordinator. In addition, results showed that agreement with the COM-B opportunity statements were higher amongst non-TDM schools. This finding may indicate that non-TDM schools may have fewer opportunities for PA, as a result of factors such as poorer facilities or locations (i.e., slippery surfaces, built urban environment, traffic hazards) that are not congruent with running a mile. Working with schools to consider how to implement TDM in schools where space/facilities are limited, and where a solution can be found could increase participation.

The third aim of this research was to assess if there was an association between school type, location, school size, deprivation measure, agreement with COM-B statements and TDM participation. A multivariate binary logistic regression model was not statistically significant indicating that these variables did not have a collective impact on whether a school was participating in TDM. We interpreted this non-significant effect as a promising finding as it suggests factors such as percentage of free school meals and deprivation measure are not barriers to participation in TDM and as intended by TDM Foundation, supporting accessibility goals. However, individual affects did show that Catholic maintained schools were almost twice as likely as controlled schools to be delivering TDM. It is unclear why uptake in Catholic schools would be higher. The results of this survey may highlight where more focused work is needed to help support schools to implement TDM.

Special Education Needs Schools who completed the survey reported more frequent participation in their own version of an ‘Active Mile’ than TDM. Furthermore, qualitative responses indicated that TDM did not meet the needs of their pupils. There is a dearth of evidence on participation in TDM amongst children with intellectual or physical disabilities to draw conclusive comparisons with the findings in our study [29, 39]. Consequently, more research is needed into the implementation and adaptation of TDM for children with special educational needs to ensure TDM is as inclusive as intended. This is particularly important as children with both physical and intellectual disabilities have an increased risk of poorer health than the general population [40] and children with physical and intellectual disabilities often face an array of additional barriers to physical activity participation, resulting in lower PA levels than their non-disabled peers [41].

### Strengths and limitations

This is the first data linkage survey to be conducted on TDM in Northern Ireland. The survey had a relatively large sample size when compared to a similar study conducted in England [32]. The survey was completed by 44.4% of primary/preparatory schools and 47.5% of special education schools. However, schools which currently take part in TDM were arguably more likely to complete the survey, resulting in a higher percentage of TDM schools represented in the sample than in Northern Ireland as a whole. However, data from The Daily Mile Foundation suggests 51% of primary schools in Northern Ireland are signed up, it is likely that some of the schools may have signed up for a one-off event and do not take part regularly, therefore this number is not much different than within the survey.

The survey was cross-sectional, therefore no cause-and-effect links can be made. The survey did not ask questions on whether teachers felt participation in TDM had any impact on other factors such a children’s fitness, behaviour, concentration, or learning time. Additionally, schools who reported doing their own version of an ‘active mile’ were not asked what this looked like or why they had chosen to participate in an ‘active mile’ rather than TDM. These factors may be important to consider in future research and help aid understanding as to why schools do or do not participate in TDM.

## Conclusions and future directions

Despite the rapid adoption of TDM over the last ten years, there is relatively little evidence surrounding the fidelity of the initiative [17]. Our cross-sectional data linkage study has gone someway to quantify participation in TDM in Northern Ireland. It was encouraging to find that over 50% of schools in Northern Ireland deliver TDM. Although the core principles state TDM is inclusive, and everyone should be able to take part, the results from this survey suggest that inclusion is not uniformly experienced in some special educational needs schools where other types of activity may be more suitable. Furthermore, this study highlighted the need to consider how the core principles of TDM could be supported further to increase levels of compliance, in frequency, to enhance the health benefits for children taking part. Overall, TDM has shown to be a worthwhile initiative for promoting PA with low costs associated with it and if supported further TDM has promise in making public health changes.

## Supporting information

S1 Checklist STROBE statement—Checklist of items that should be included in reports of observational studies. (DOCX)

## References

[1] Biddle SJ, Ciaccioni S, Thomas G, Vergeer I. Physical activity and mental health in children and adolescents: An updated review of reviews and an analysis of causality. Psychology of sport and exercise. 2019 May 1;42:146–55.

[2] Jago R, Salway R, Emm-Collison L, Sebire SJ, Thompson JL, Lawlor DA. Association of BMI category with change in children’s physical activity between ages 6 and 11 years: A longitudinal study. International journal of obesity. 2020 Jan 1;44(1):104–13. doi: 10.1038/s41366-019-0459-0 31712707 PMC6923172

[3] Eddolls WT, McNarry MA, Stratton G, Winn CO, Mackintosh KA. High-intensity interval training interventions in children and adolescents: a systematic review. Sports medicine. 2017 Nov;47:2363–74. doi: 10.1007/s40279-017-0753-8 28643209 PMC5633633

[4] Rodriguez-Ayllon M, Cadenas-Sánchez C, Estévez-López F, Muñoz NE, Mora-Gonzalez J, Migueles JH, et al. Role of physical activity and sedentary behavior in the mental health of preschoolers, children and adolescents: a systematic review and meta-analysis. Sports medicine. 2019 Sep;49(9):1383–410. doi: 10.1007/s40279-019-01099-5 30993594

[5] Department of Health and Social Care. Physical Activity Guidelines: UK Chief Medical Officers’ Report. Department of Health and Social Care. 2019: https://assets.publishing.service.gov.uk/government/uploads/system/uploads/attachment_data/file/832868/uk-chief-medical-officers-physical-activity-guidelines.pdf

[6] Sport England. Active Lives Children and Young People Survey: Academic year 2020–21. 2021. https://sportengland-production-files.s3.eu-west-2.amazonaws.com/s3fs-public/2021-12/Active%20Lives%20Children%20and%20Young%20People%20Survey%20Academic%20Year%202020-21%20Report.pdf?VersionId=3jpdwfbsWB4PNtKJGxwbyu5Y2nuRFMBV.

[7] Connolly S, Carlin A, Johnston A, Woods C, Powell C, Belton S, et al. Physical activity, sport and physical education in Northern Ireland school children: A cross-sectional study. International journal of environmental research and public health. 2020 Sep;17(18):6849. doi: 10.3390/ijerph17186849 32961784 PMC7559058

[8] O’Brien W, Belton S, Fitzpatrick B, Shannon S, Brennan D, Chambers F et al. Relationship between gender, physical activity, screen time, body mass index and wellbeing in Irish children from social-disadvantage. Child Care in Practice. 2021; 1–15.

[9] Sport England. Active Lives Children and Young People Survey. Academic Year 2021–22. 2022. https://sportengland-production-files.s3.eu-west-2.amazonaws.com/s3fs-public/2022-12/Active%20Lives%20Children%20and%20Young%20People%20Survey%20Academic%20Year%202021-22%20Report.pdf?VersionId=R5_hmJHw5M4yKFsewm2vGDMRGHWW7q3E

[10] Public Health England. What works in schools and colleges to increase physical activity? A resource for head teachers, college principals, staff working in education settings, school nurses, directors of public health, Active Partnerships and wider partners. 2020. https://assets.publishing.service.gov.uk/government/uploads/system/uploads/attachment_data/file/876242/Guidance_to_increase_physical_activity_among_children_and_young_people_in_schools_and_colleges.pdf

[11] The Daily Mile Foundation. The Daily Mile. 2022: https://thedailymile.co.uk

[12] Venkatraman T, Honeyford K, Costelloe CE, Bina R, van Sluijs EM, Viner RM, et al. Sociodemographic profiles, educational attainment and physical activity associated with the daily Mile™ registration in primary schools in England: a national cross-sectional linkage study. J Epidemiol Community Health. 2021 Feb 1;75(2):137–44. 33004657 10.1136/jech-2020-214203 PMC7815899

[13] Ferry F, Bunting B, Murphy S, O’Neill S, Stein D, Koenen K. Traumatic events and their relative PTSD burden in Northern Ireland: a consideration of the impact of the ‘Troubles’. Social psychiatry and psychiatric epidemiology. 2014 Mar;49:435–46. 23959590 10.1007/s00127-013-0757-0

[14] O’Neill S, Ferry F, Heenan D. Mental health disorders in Northern Ireland: the economic imperative. The Lancet Psychiatry. 2016 May 1;3(5):398–400. doi: 10.1016/S2215-0366(16)00084-5 27155507

[15] Griffin N. How the lack of government is affecting healthcare in Northern Ireland. BMJ: British Medical Journal. 2019 Jan 14;364.

[16] McMurray, S. Physical Activity and the Wellbeing of Children and Young People. Research and Information Service Briefing Paper. 2020. http://www.niassembly.gov.uk/globalassets/documents/raise/publications/2017-2022/2021/education/4021.pdf

[17] Breslin G, Hillyard M, Brick N, Shannon S, McKay-Redmond B, McConnell B. A systematic review of the effect of The Daily Mile^™^ on children’s physical activity, physical health, mental health, wellbeing, academic performance and cognitive function. Plos one. 2023 Jan 12;18(1):e0277375. 36634113 10.1371/journal.pone.0277375 PMC9836306

[18] Brustio PR, Mulasso A, Lupo C, Massasso A, Rainoldi A, Boccia G. The Daily Mile is able to improve cardiorespiratory fitness when practiced three times a week. International Journal of Environmental Research and Public Health. 2020;17(6):2095. doi: 10.3390/ijerph17062095 . 32235688 PMC7143074

[19] Lee Y, Park S. Understanding of physical activity in social ecological perspective: application of multilevel model. Frontiers in Psychology. 2021 Mar 5;12:622929. doi: 10.3389/fpsyg.2021.622929 33746840 PMC7973361

[20] Biddle SJ, Gorely T, Faulkner G, Mutrie N. Psychology of physical activity: a 30-year reflection on correlates, barriers, and theory. International Journal of Sport and Exercise Psychology. 2023 Jan 2;21(1):1–4.

[21] Breheny K, Passmore S, Adab P, Martin J, Hemming K, Lancashire ER, et al. Effectiveness and cost-effectiveness of The Daily Mile on childhood weight outcomes and wellbeing: a cluster randomised controlled trial. International Journal of Obesity. 2020;44(4):812–22. doi: 10.1038/s41366-019-0511-0 . 31988481 PMC7101281

[22] Brustio PR, Mulasso A, Marasso D, Ruffa C, Ballatore A, Moisè P et al. The Daily Mile: 15 minutes running improves the physical fitness of Italian primary school children. International Journal of Environmental Research and Public Health. 2019; 16(20):3921. doi: 10.3390/ijerph16203921 . 31618975 PMC6843651

[23] Morris JL, Daly-Smith A, Archbold VS, Wilkins EL, McKenna J. The Daily Mile™ initiative: Exploring physical activity and the acute effects on executive function and academic performance in primary school children. Psychology of Sport and Exercise. 2019;45:101583.

[24] Arkesteyn A, Vancampfort D, Firth J, Van Damme T. Mental health outcomes of the Daily Mile in elementary school children: a single‐arm pilot study. Child and Adolescent Mental Health. 2022 Jun 24. 10.1111/camh.12573 35748760

[25] Ryde GC, Booth JN, Brooks NE, Chesham RA, Moran CN, Gorely T. The Daily Mile: What factors are associated with its implementation success? PloS one. 2018; 13(10): e0204988. doi: 10.1371/journal.pone.0204988 . 30286175 PMC6171888

[26] Michie S, Van Stralen MM, West R. The behaviour change wheel: a new method for characterising and designing behaviour change interventions. Implementation science. 2011 Dec 1;6(1):42. doi: 10.1186/1748-5908-6-42 . 21513547 PMC3096582

[27] Department of Education. Newcomer- Guidelines for Schools. Criteria for Designating a Pupil as Newcomer and Sharing Good Practice. 2010. https://www.education-ni.gov.uk/sites/default/files/publications/de/newcomer-guidelines-for-schools.pdf

[28] Northern Ireland Statistics and Research Agency (NISRA) Census. 2021. https://www.nisra.gov.uk/statistics/census/2021-census

[29] McDermott G, Brick NE, Shannon S, Fitzpatrick B, Taggart L. Barriers and facilitators of physical activity in adolescents with intellectual disabilities: An analysis informed by the COM‐B model. Journal of Applied Research in Intellectual Disabilities. 2022 May;35(3):800–25. doi: 10.1111/jar.12985 35229409 PMC9305883

[30] Northern Ireland Statistics and Research Agency (NISRA) Northern Ireland Multiple Deprivation Measure 2017. https://www.nisra.gov.uk/statistics/deprivation/northern-ireland-multiple-deprivation-measure-2017-nimdm2017

[31] Department of Education. School Enrolments- Northern Ireland summary data. 2022. https://www.education-ni.gov.uk/publications/school-enrolments-northern-ireland-summary-data

[32] Routen A, Aguado MG, O’Connell S, Harrington D. The Daily Mile in practice: implementation and adaptation of the school running programme in a multiethnic city in the UK. BMJ open. 2021; 11(8): e046655. doi: 10.1136/bmjopen-2020-046655 . 34341042 PMC8330578

[33] Hanckel B, Ruta D, Scott G, Peacock JL, Green J. The Daily Mile as a public health intervention: a rapid ethnographic assessment of uptake and implementation in South London, UK. BMC Public Health. 2019 Dec;19(1):1–4. 31455316 10.1186/s12889-019-7511-9 PMC6712825

[34] Larson TA, Normand MP, Morley AJ, Miller BG. A functional analysis of moderate‐to‐vigorous physical activity in young children. Journal of Applied Behavior Analysis. 2013 Mar;46(1):199–207. doi: 10.1002/jaba.8 24114094

[35] Ekkekakis P, Brand R. Affective responses to and automatic affective valuations of physical activity: Fifty years of progress on the seminal question in exercise psychology. Psychology of Sport and Exercise. 2019 May 1;42:130–7.

[36] Teychenne M, White RL, Richards J, Schuch FB, Rosenbaum S, Bennie JA. Do we need physical activity guidelines for mental health: What does the evidence tell us?. Mental health and physical activity. 2020 Mar 1;18:100315.

[37] Malden S, Doi L. The Daily Mile: teachers’ perspectives of the barriers and facilitators to the delivery of a school-based physical activity intervention. BMJ open. 2019 Mar 1;9(3):e027169. doi: 10.1136/bmjopen-2018-027169 30837259 PMC6429867

[38] Marchant E, Todd C, Stratton G, Brophy S. The Daily Mile: Whole-school recommendations for implementation and sustainability. A mixed-methods study. PloS one. 2020 Feb 5;15(2):e0228149. doi: 10.1371/journal.pone.0228149 32023297 PMC7001902

[39] Fitzpatrick B, Taggart L, Breslin G. Developing a School Based Physical Activity Protocol for those with Intellectual Disability. In: Cotterill S, Weston N, Breslin B, Editors. Sport and Exercise Psychology: Practitioner Case Studies. John Wiley & Sons.2016. p.0-520.

[40] Taggart L, Johnston A, Mullhall P, Hassiotis A, Murphy M, Slater P, et al. ‘Walk Buds’: A walking programme to increase physical activity, physical fitness and emotional wellbeing, in 9–13 yr old children with intellectual disability. A study protocol for a clustered RCT. Contemporary Clinical Trials. 2022 Aug 1;119:106856. 35863694 10.1016/j.cct.2022.106856

[41] Einarsson IÓ, Ólafsson Á, Hinriksdóttir G, Jóhannsson E, Daly D, Arngrímsson SA. Differences in physical activity among youth with and without intellectual disability. Med Sci Sports Exerc. 2015 Feb 1;47(2):411–8. doi: 10.1249/MSS.0000000000000412 24983335

