# Supplementary material for: Predicting school uptake of The Daily Mile in Northern Ireland- a data linkage study with School Census Data and Multiple Deprivation Measures
Source: PLoS One. 2023 Dec 14;18(12):e0294648. doi: 10.1371/journal.pone.0294648 (PMC10721005; doi:10.1371/journal.pone.0294648)
Supplement: S1 Checklist — (DOCX) [file pone.0294648.s001.docx]

STROBE Statement—checklist of items that should be included in reports of observational studies

|  | Item No. | Recommendation | Page  No. | Relevant text from manuscript |
| --- | --- | --- | --- | --- |
| **Title and abstract** | 1 | (*a*) Indicate the study’s design with a commonly used term in the title or the abstract | 2 | “online cross-sectional survey” |
|  |  | (*b*) Provide in the abstract an informative and balanced summary of what was done and what was found | 2-3 | See Methods sections and Results-Online survey sent to all primary and special education schools in NI. Survey data linked to NI Multiple Deprivation Measure and School census data. Over half (54.7%) of primary schools and 36.8% of special education schools reported taking part in TDM. Wide variation in adherence to TDM core principles. No assocaiton found between deprivation and TDM uptake. |
| Introduction | | | |  |
| Background/rationale | 2 | Explain the scientific background and rationale for the investigation being reported | 4-8 | Low PA levels in children, need for interventions to increase PA. TDM programme widely adopted worldwide- unclear how many children in NI participate and if carried out in line with core principles. NI region with complex social, political and economic circumstances. Need to understand uptake of TDM in NI. |
| Objectives | 3 | State specific objectives, including any prespecified hypotheses | 7-8 | 1. To conduct a cross-sectional suvey of primary and special educational needs schools to determine the prevalence of TDM in NI. 2. Assess adherence to TDM core principles. 3. To explore predictors of TDM uptake. |
| Methods | | | |  |
| Study design | 4 | Present key elements of study design early in the paper 8-9 |  | Cross-sectional survey sent to all primary and special education schools in NI. Survey |
| Setting | 5 | Describe the setting, locations, and relevant dates, including periods of recruitment, exposure, follow-up, and data collection | 8-9 | Details of NI given and school types. Survey platform Qualtrics, survey available for online completion from 31^st^ August 2022 until 16^th^ December 2022. |
| Participants | 6 | (*a*) *Cohort study*—Give the eligibility criteria, and the sources and methods of selection of participants. Describe methods of follow-up  *Case-control study*—Give the eligibility criteria, and the sources and methods of case ascertainment and control selection. Give the rationale for the choice of cases and controls  *Cross-sectional study*—Give the eligibility criteria, and the sources and methods of selection of participants | 8-9 | Cross-sectional study- all primary and special education schools in NI were eligible. School Principal or teacher with most knowledge on school’s participation in TDM asked to complete survey. |
|  |  | (*b*) *Cohort study*—For matched studies, give matching criteria and number of exposed and unexposed  *Case-control study*—For matched studies, give matching criteria and the number of controls per case |  |  |
| Variables | 7 | Clearly define all outcomes, exposures, predictors, potential confounders, and effect modifiers. Give diagnostic criteria, if applicable | 10-11 | All measures given- school demographics, TDM participation, assessment of core principles. Barriers to participation. |
| Data sources/ measurement | 8* | For each variable of interest, give sources of data and details of methods of assessment (measurement). Describe comparability of assessment methods if there is more than one group | 10-11 | Details of survey, School Census Data and NI Multiple Deprivation measure given. |
| Bias | 9 | Describe any efforts to address potential sources of bias |  |  |
| Study size | 10 | Explain how the study size was arrived at |  | Cross-sectional survey sent to all primary and special education schools in NI |

Continued on next page

| Quantitative variables | 11 | Explain how quantitative variables were handled in the analyses. If applicable, describe which groupings were chosen and why | 12-13 | Schools were categorized as TDM schools or non TDM schools. |
| --- | --- | --- | --- | --- |
| Statistical methods | 12 | (*a*) Describe all statistical methods, including those used to control for confounding | 12-13 | Details of statistical analyses give- i.e. frequencies and percentages for categorical variables, means and SD for continuous variables. Chi squared tests to test for associations and multivariate binary logistic regression to determine if there were any associations between variables and TDM participation |
|  |  | (*b*) Describe any methods used to examine subgroups and interactions |  |  |
|  |  | (*c*) Explain how missing data were addressed |  |  |
|  |  | (*d*) *Cohort study*—If applicable, explain how loss to follow-up was addressed  *Case-control study*—If applicable, explain how matching of cases and controls was addressed  *Cross-sectional study*—If applicable, describe analytical methods taking account of sampling strategy |  |  |
|  |  | (*e*) Describe any sensitivity analyses |  |  |
| Results | | | | |
| Participants | 13* | (a) Report numbers of individuals at each stage of study—eg numbers potentially eligible, examined for eligibility, confirmed eligible, included in the study, completing follow-up, and analysed |  | 13 Details on  numbers at each stage given in results |
|  |  | (b) Give reasons for non-participation at each stage |  | 13 |
|  |  | (c) Consider use of a flow diagram |  | Not necessary |
| Descriptive data | 14* | (a) Give characteristics of study participants (eg demographic, clinical, social) and information on exposures and potential confounders |  | 14-15 Demographic details of schools given |
|  |  | (b) Indicate number of participants with missing data for each variable of interest |  |  |
|  |  | (c) *Cohort study*—Summarise follow-up time (eg, average and total amount) |  |  |
| Outcome data | 15* | *Cohort study*—Report numbers of outcome events or summary measures over time |  |  |
|  |  | *Case-control study—*Report numbers in each exposure category, or summary measures of exposure |  |  |
|  |  | *Cross-sectional study—*Report numbers of outcome events or summary measures |  |  |
| Main results | 16 | (*a*) Give unadjusted estimates and, if applicable, confounder-adjusted estimates and their precision (eg, 95% confidence interval). Make clear which confounders were adjusted for and why they were included |  |  |
|  |  | (*b*) Report category boundaries when continuous variables were categorized |  |  |
|  |  | (*c*) If relevant, consider translating estimates of relative risk into absolute risk for a meaningful time period |  |  |

Continued on next page

| Other analyses | 17 | Report other analyses done—eg analyses of subgroups and interactions, and sensitivity analyses |  |  |
| --- | --- | --- | --- | --- |
| Discussion | | | | |
| Key results | 18 | Summarise key results with reference to study objectives | 21-26 |  |
| Limitations | 19 | Discuss limitations of the study, taking into account sources of potential bias or imprecision. Discuss both direction and magnitude of any potential bias | 26 |  |
| Interpretation | 20 | Give a cautious overall interpretation of results considering objectives, limitations, multiplicity of analyses, results from similar studies, and other relevant evidence | 21-26 |  |
| Generalisability | 21 | Discuss the generalisability (external validity) of the study results | 21-26 |  |
| Other information | |  | | |
| Funding | 22 | Give the source of funding and the role of the funders for the present study and, if applicable, for the original study on which the present article is based | Given |  |

*Give information separately for cases and controls in case-control studies and, if applicable, for exposed and unexposed groups in cohort and cross-sectional studies.

**Note:** An Explanation and Elaboration article discusses each checklist item and gives methodological background and published examples of transparent reporting. The STROBE checklist is best used in conjunction with this article (freely available on the Web sites of PLoS Medicine at http://www.plosmedicine.org/, Annals of Internal Medicine at http://www.annals.org/, and Epidemiology at http://www.epidem.com/). Information on the STROBE Initiative is available at www.strobe-statement.org.
